# EmoWELL: effectiveness of a serious game for emotion regulation in emerging adulthood

**DOI:** 10.3389/fpsyg.2025.1561418

**Published:** 2025-04-15

**Authors:** Saray Velert-Jiménez, Selene Valero-Moreno, José-Antonio Gil-Gómez, Marián Pérez-Marín, Inmaculada Montoya-Castilla

**Affiliations:** ^1^Department of Personality, Assessment and Psychological Treatments, Faculty of Psychology and Speech Therapy, Universitat de València, València, Spain; ^2^University Institute of Automatics and Industrial Informatics, Universitat Politècnica de València, València, Spain

**Keywords:** serious game, emerging adulthood, emotion regulation, emotion dysregulation, psychological well-being

## Abstract

**Introduction:**

Emerging adulthood represents a critical period for developing emotion regulation skills, as individuals face new roles and responsibilities while often employing maladaptive regulatory strategies. Given the increasing use of technology among emerging adults, this study evaluated emoWELL, a serious game designed to enhance emotion regulation during this developmental stage.

**Methods:**

Using a quasi-experimental design, 114 university students in emerging adulthood (aged 18–25) were assigned to either an experimental group using emoWELL or a control group. Analyses included MANOVA, MANCOVA, hierarchical multiple regression, Reliable Change Index (RCI), moderation, mediation, and qualitative analyses of participants’ feedback.

**Results:**

Significant improvements were observed in the experimental group (Wilks’ *λ* = .68; *F* = 2.59; *p* = 0.003; *η*^2^ = .32), particularly in reducing expressive suppression strategy, emotional rejection, lack of emotional control, and overall emotion dysregulation. Mediation analyses revealed that enhanced emotion regulation indirectly improved self-acceptance and environmental mastery. Moderation analyses indicated that baseline anxiety and depression levels influenced the intervention’s effectiveness, with fewer symptom levels associated with better emotional outcomes post-intervention. The game received positive user feedback, particularly regarding its psychoeducational content and emotional awareness components.

**Discussion:**

While emoWELL shows promise as a preventive tool for emotion regulation in emerging adults with low emotional symptomatology, its effectiveness may be limited for those with elevated anxiety and depression levels, suggesting its optimal use as a complementary tool under professional supervision or in university settings with appropriate support.

## 1 Introduction

The period of emerging adulthood, which typically spans the ages of 18 to 29, represents a distinct developmental stage that differs from both adolescence and young adulthood, characterized by unique demographic patterns and subjective experiences (Arnett, 2000; Arnett, 2015). During this period, individuals typically develop emotion regulation skills that encompass emotional, cognitive, and behavioral domains (Arnett, 2014). Emotion regulation can be defined as the modification of which emotions are experienced, when they occur, and how they are felt or expressed (Gross, 1998b). According to the modal model of emotions (Gross, 1998a), emotions arise from person–situation interactions that capture attention, hold relevance to an individual’s goals, and produce coordinated, flexible multisystem responses. These responses, in turn, shape subsequent interactions, creating a dynamic, ongoing process. Expanding upon this base, Gross’s emotion regulation process model (Gross, 1998b) outlines five categories of emotion regulation strategies processes: situation selection, situation modification, attentional deployment, cognitive change, and response modulation. These families correspond to specific stages in the emotion-generative sequence, which offers distinct opportunities for regulation. For example, situation selection involves choosing environments to encourage or avoid certain emotions, while situation modification alters the situation’s emotional impact. Attentional deployment redirects focus within a situation, and cognitive change reinterprets its significance, with reappraisal being a widely studied example. Finally, response modulation acts on emotional responses after they arise, such as expressive suppression, which inhibits outward emotional expressions. This model also emphasizes the dynamic, recursive nature of emotion regulation, as emotional responses feed back into the person–situation interaction, influencing future emotional experiences. Along these lines, other authors also emphasize that emotion regulation comprises being aware of one’s emotions, understanding them, accepting them, controlling impulsive actions, and acting in alignment with desired objectives when facing negative emotions. It also includes the capacity to adaptively use appropriate strategies to adjust emotional reactions as needed to meet personal goals and situational requirements. Difficulties in emotion regulation, i.e., the absence of these skills, is known as emotional dysregulation (Gratz and Roemer, 2004).

The capacity for healthy emotional functioning is of particular importance to this population, as they assume new roles, consolidate their identities, and confront greater responsibilities (Bonnie et al., 2015; Thomas and Zolkoski, 2020; Zhu et al., 2024). Nevertheless, John and Gross (2004) identified age-related differences in the regulation of emotions, noting that younger adults tend to employ more maladaptive strategies, such as expression suppression, in comparison to older adults. Emerging adulthood is a fundamental phase for the development and consolidation of emotion regulation abilities, as the patterns established during this stage often have long-lasting effects throughout adulthood (Schulenberg et al., 2004; Zimmermann and Iwanski, 2014).

Emotion regulation is also an important treatment target for this population due to the broad impact of emotion difficulties, particularly given that expressive suppression can adversely affect various well-being indicators (Mouatsou and Koutra, 2021). For example, cognitive reappraisal appears to have a positive effect on life satisfaction, the tendency to perceive events more positively, and overall well-being (Cheung et al., 2021; Newman and Nezlek, 2022). For emerging adults specifically, cognitive reappraisal serves as a protective factor for psychological health (Chen and Cheung, 2021). It seems to enhance resilience, with self-esteem partially mediating this relationship (Mouatsou and Koutra, 2021), and to contribute to healthier social and family functioning (Medellu and Azzahra, 2022). Neuroimaging studies suggest that cognitive reappraisal not only reduces immediate emotional distress but may also have lasting effects on emotional reactivity to repeated stressors (Willner et al., 2022). Moreover, habitual use of cognitive reappraisal can protect against depressive symptoms, especially in the context of stressful life events (Roos and Bennett, 2023), making it particularly valuable as emerging adults navigate the various challenges and transitions characteristic of this life stage. In contrast, expressive suppression seems to be associated with poorer general health, a tendency to interpret events negatively, and lower well-being; while emotion regulation difficulties appear to be linked to higher levels of insomnia, depression, lower resilience, and poorer well-being and mental-health (Cheung et al., 2021; Newman and Nezlek, 2022; Rufino et al., 2022; Zagaria et al., 2023).

Paradoxically, there are factors that may limit the improvement of emotion regulation, such as participants’ prior expectations of improvement (Long et al., 2020) or difficulties in cognitive and executive functioning (Molavi et al., 2020). Executive functions, including planning, decision-making, problem-solving, and inhibitory control, are essential for managing emotions effectively (Fernandes et al., 2023). These cognitive processes enable individuals to evaluate emotional stimuli, select appropriate responses, and inhibit impulsive reactions that might exacerbate emotional distress (Stubberud et al., 2020). When these cognitive and executive functions are well-developed, individuals may be able to employ more effective emotion regulation strategies, potentially leading to better mental health outcomes. Conversely, impairments in these domains could contribute to the use of maladaptive emotion regulation strategies, such as catastrophizing or avoidance, which might exacerbate emotional difficulties (Fernandes et al., 2023). In this context, the literature suggests that psychological disorders often involve rigid responses to environmental demands, which may hinder adaptive emotion regulation (Aldao, 2013). For example, Visted et al. (2018) found that individuals with Major Depressive Disorder frequently employed maladaptive strategies, such as rumination, which may hamper the improvement of emotion regulation. Similarly, Post-Traumatic Stress Disorder seems to be associated with high levels of rumination, thought suppression, and experiential avoidance, which may lead to greater emotion dysregulation (Seligowski et al., 2015). While these studies have not been conducted specifically in emerging adults, this suggests that emotional symptoms such as anxiety and depression might interfere with one’s ability to benefit from interventions aimed at improving emotion regulation. Further research in this population could help clarify the relationship between emotion regulation and these specific symptoms.

Regarding the emotion regulation relation with well-being, various studies on emerging adults also highlight the importance of an adequate psychological well-being in relation to mental health (e.g., (Leustek and Theiss, 2020; Lucas et al., 2024). Psychological well-being is a multidimensional construct that encompasses affective satisfaction and the fulfillment of deeper human potentials. Unlike traditional approaches to well-being, which often emphasize transient happiness or life satisfaction, psychological well-being represents a holistic framework that integrates enduring challenges and achievements essential for personal growth and meaningful living (Ryff, 1989b,c). Specifically, is operationalized through six core dimensions that capture the richness of human psychological functioning (Ryff, 1989b): self-acceptance (a positive attitude toward oneself, characterized by the acknowledgment and acceptance of personal strengths and weaknesses), environmental mastery (the ability to effectively manage one’s life and surrounding environment to fulfill personal needs and goals), positive relations with others (the capacity to form deep and trusting relationships), autonomy (the ability to maintain independence in decision-making, resisting external pressures to conform), purpose in life (a sense of direction and meaning, based on long-term objectives that provide significance to one’s existence) and personal growth (a dynamic sense of self-improvement, openness and the realization of one’s potential). Therefore, it seems necessary that emerging adults develop a functional emotion regulation, being a critical skill for a healthy development which can help to prevent a range of negative outcomes (Eisenberg and Spinrad, 2004; Gatto et al., 2022).

Psychological interventions for emotion regulation during emerging adulthood have shown beneficial outcomes (Cheung et al., 2021; Newman and Nezlek, 2022). Emerging adults have grown up with technology as an integral part of their lives, influencing in areas such as their learning, social interactions, or identity formation (Markoc, 2025; Munshi et al., 2024). Given that technology-based interventions have shown positive effects (Coifman and Gunstad, 2024) and the high prevalence of video game use during this developmental period (Micallef et al., 2021), digital platforms present a particularly relevant approach for this population. In this sense, serious games are simulation software designed to help people learn practical concepts, improve knowledge and skills in areas such as health, wellness, education, among others (Din et al., 2023). Specifically, regarding the emerging adult population, various serious games have demonstrated their utility across different domains. These include applications in substance use prevention and intervention (Martínez-Miranda and Espinosa-Curiel, 2022), extensions of mental health support services that improve engagement and therapeutic efficacy (Townsend et al., 2022), and enhancements to learning outcomes and skill development (Connolly et al., 2012; Wouters et al., 2013), between others. In recent years, more serious games have been developed to address emotion regulation (e.g., 44–46). However, to our knowledge, no serious games specifically target emerging adults, considering the unique characteristics and challenges of this developmental stage. Although these interventions look promising, more research is still needed to see how effective they can be on the mental health of emerging adults (Vié et al., 2024).

Building on the aforementioned background, the present study aimed to evaluate the effectiveness of an emotion regulation serious game designed for emerging adults: emoWELL. Specifically, the program was expected to enhance emotion regulation strategies and subsequently improve psychological well-being. Based on prior research, the study proposed three primary hypotheses: (1) the program is expected to enhance emotion regulation, specifically by reducing emotion dysregulation and expressive suppression, along with an improvement in cognitive reappraisal; (2) psychological well-being will improve indirectly through the strengthening of emotion regulation; (3) baseline levels of emotional symptoms, specifically anxiety and depression, will moderate the program’s effects, such that participants with higher symptom levels will show less improvement in emotion regulation.

## 2 Materials and methods

### 2.1 Participants

Participants were included in the study if they met the following inclusion criteria: (a) being within the emerging adulthood age range (18–29 years), (b) completing at least 80% of the survey, (c) not responding randomly, as determined by the Oviedo Infrequency Scale (Fonseca-Pedrero et al., 2009), (d) completing the intervention program, and (e) not being currently enrolled in another emotion regulation program. Therefore, participants were excluded from the study if they met any of the following criteria: (a) being younger than 18 or older than 29 years, (b) completing less than 80% of the survey, (c) showing random response patterns as identified by the Oviedo Infrequency Scale (Fonseca-Pedrero et al., 2009), (d) not completing the intervention program, or (e) being currently enrolled in another emotion regulation program.

**Figure 1:**
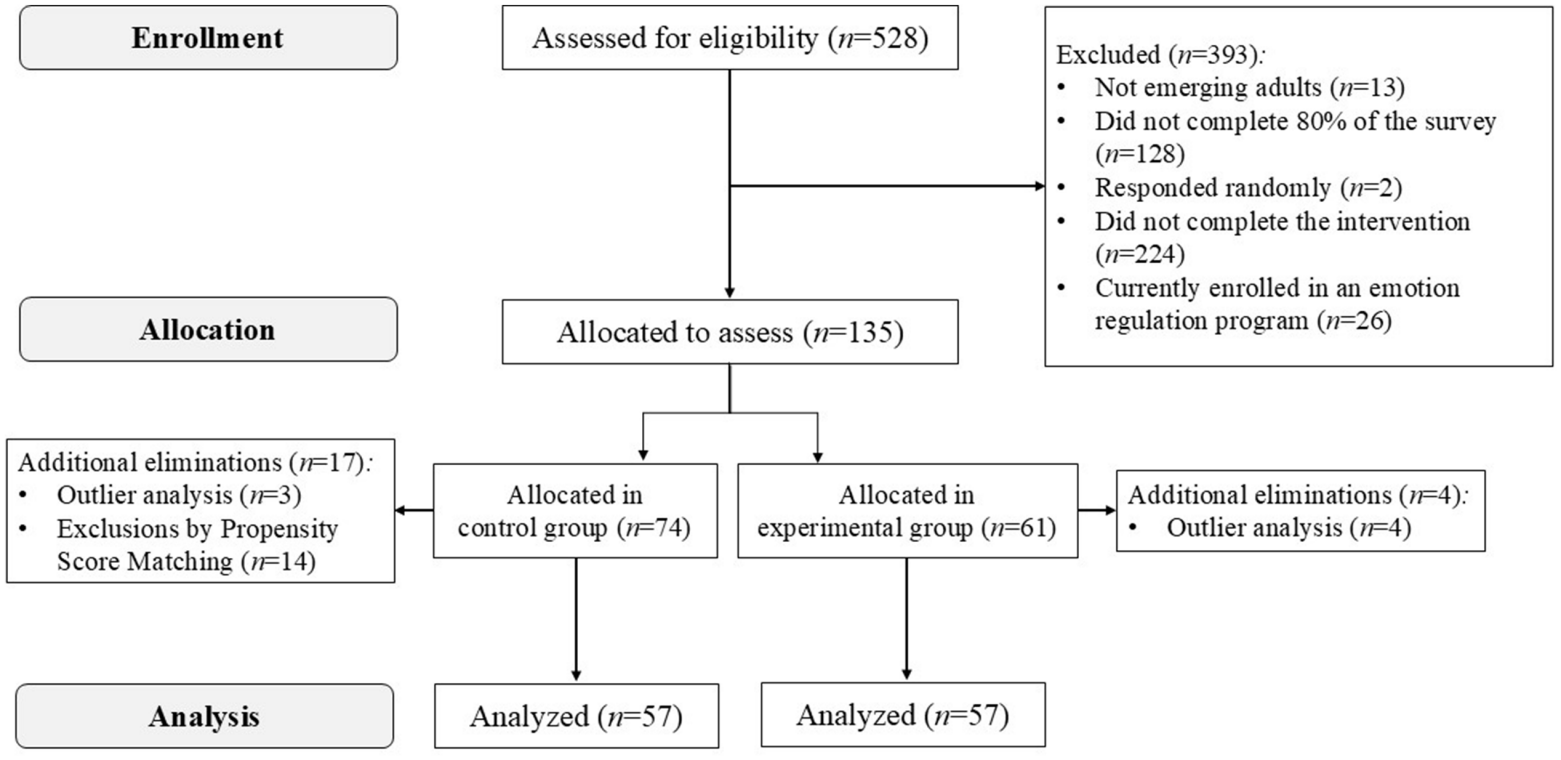
Flow diagram of study participants.

In the initial database, there were a total of 528 participants. After applying the five inclusion and exclusion criteria, 393 participants were excluded. Of the remaining 135 participants, differences in pre-intervention measures were observed between the control and experimental groups, as will be discussed in more detail later. Consequently, 7 participants with outlier values, based on Mahalanobis distance, were removed. Due to the differences found, using Propensity Score Matching, an additional 14 participants were excluded. Ultimately, the final sample consisted of 114 participants, evenly distributed between the experimental group (57 participants) and the control group (57 participants). Figure 1 illustrates the participant’s flow in greater detail.

The participants were university students. Of the final sample, 86.8% identified as women and 13.2% as men. Participants were aged between 18 and 25 years (*M* = 19.95, SD = 1.41) and were evenly distributed between the control and experimental groups, with 50% in each group. The majority of participants lived with their family of origin (e.g., parents, legal guardians) (62.2%), while 35.10% lived in shared housing with multiple people, and 2.70% lived alone, with a partner, or with their nuclear family (e.g., children). Most participants reported being financially dependent on their family (61.40%), did not have any diagnosed physical or psychological conditions (88.60%), and were not attending psychological therapy (85.10%). Additionally, 54.40% reported being in a stable romantic relationship, while 45.60% were not.

### 2.2 Procedure

The study followed an analytical design and adhered to the recommendations of the Declaration of Helsinki (World Medical Association Declaration of Helsinki, 2013). The University Ethics Committee approved the study (registration number 2013883). emoWELL serious game has been registered with the following intellectual property number: UV-SW-202460R, and is listed in ClinicalTrials.gov under the identifier NCT06049407.

This study used a quasi-experimental design for both groups. Members of the research team contacted faculty interested in disseminating the intervention platform to students and recruiting participants. Participation was voluntary, and students who chose to enroll were then assigned to either the experimental or control group. Faculty explained to interested university students that the study involved a technology platform for emerging adults, provided the survey links, shared the serious game download file, and reminded students of the study start and end dates. Participants completed the first set of pre-intervention questionnaires in February 2024 and completed the study by early May of that year. They engaged with the platform independently from home, outside of their academic or work hours, between the two measures.

### 2.3 Instruments

Regarding sociodemographic variables, gender, age, number of household members, perceived economic autonomy, current occupation, relationship status, possible psychological or physical difficulty diagnosed, as well as attendance in current psychological therapy, were assessed using *ad hoc* questions.

Emotion regulation was assessed using the Emotion Regulation Questionnaire (ERQ) (Cabello et al., 2013; Gross and John, 2003) and the Difficulties in Emotion Regulation Scale (DERS) (Gratz and Roemer, 2004; Hervás and Jódar, 2008). Consistent with the original version, the Spanish validation of the ERQ (Cabello et al., 2013) comprises 10 items rated on a 7-point Likert scale (1 = Strongly disagree, 7 = Strongly agree). The items are divided into two dimensions: cognitive reappraisal and expressive suppression. In the original study by Gross and John (2003), the Cronbach’s *α* values for the two subscales ranged from 0.75 to 0.82 for the reappraisal items and from 0.68 to 0.76 for the suppression items across four different samples. In the current study, Cronbach’s alpha values for the reappraisal subscale were 0.76 in the initial assessment and 0.74 in the post-intervention assessment, while for the suppression subscale, they were 0.83 in the initial assessment and 0.85 in the post-intervention assessment. The DERS Spanish version (Hervás and Jódar, 2008) assess emotion dysregulation and consists of 28 items on a Likert scale ranging from 1 (Almost never) to 5 (Almost always). The five dimensions of the scale are lack of emotional attention, emotional confusion, emotional rejection, emotion life interference, and emotional lack of control, along with an overall global scale. The internal consistency of the Spanish validation was comparable to that of the original study, yielding an alpha coefficient of 0.93 for both versions. In the current study, Cronbach’s alpha values for the DERS scale ranged from 0.80 to 0.90 (0.93 in the case of the global scale) in the initial assessment, ranging from 0.85 to 0.92 (0.95 in the global scale) in the post-intervention assessment.

Psychological well-being was evaluated through the Scales of Psychological Well-being (Ryff, 1989a). The instrument comprises 29 items categorized into six dimensions: self-acceptance, positive relations with others, autonomy, environmental mastery, purpose in life, and personal growth. In this research, the 29-item version (Díaz et al., 2006; Van Dierendonck, 2004) was used, with responses gathered on a Likert scale ranging from 1 (Totally Disagree) to 6 (Totally Agree). The scales showed sufficient internal consistency in both the original version (with *α* values ranging from 0.78 to 0.81) and the Spanish validation (*α* = 0.70–0.84). In this study, Cronbach’s alpha values ranged from 0.71 to 0.87 in the initial assessment, ranging from 0.70 to 0.86 in the post-intervention assessment.

Depression and anxiety symptoms were assessed using the Brief Symptom Inventory-18 (BSI-18) (Derogatis, 2001; Derogatis, 2013). The instrument consists of 18 items answered on a five-point Likert scale ranging from 0 (not at all) to 4 (extremely). The instrument provides three symptom scores (depression, anxiety, and somatization) and a total score. Regarding the scales used in this study, they showed an accurate reliability in the original version (*α* values = 0.84 for depression, 0.79 for anxiety) (Derogatis, 2001) and in the Spanish version (*α* values = 0.84 for depression, 0.81 for anxiety) (Derogatis, 2013). In the present study, the Cronbach’s alpha values were 0.84 for depression and 0.77 for anxiety at baseline, and 0.87 for depression and 0.80 for anxiety at post-intervention.

Finally, two further *ad hoc* questions were asked to the experimental group to assess their opinion of the intervention. The first was a Likert question (How much did you like emoWELL?), rated on a scale of 1 to 5 (1 = “I did not like it at all”; 5 = “I liked it a lot”). The second was an open question (What did you like most about emoWELL?), designed to elicit spontaneous responses, allowing for the exploration of possible psychological content as well as motivational or engagement elements.

### 2.4 Intervention program

EmoWELL is a serious computer game designed to enhance emotion regulation in emerging adults. The intervention is based on the process model of emotion regulation proposed by Gross (1998b), focusing on fostering healthy emotion regulation strategies and addressing the instrumental role of emotions. The game employs engaging storytelling and interactive exercises to guide players through various scenarios, each representing key areas of emotion regulation, such as managing stress, improving self-esteem, reducing perceived loneliness, and promoting psychological well-being.

The narrative unfolds as the player embarks on a train journey, visiting different zones where they encounter diverse challenges and opportunities to practice adaptive emotion regulation strategies. These activities aim to help players reflect on their current emotional habits, understand the consequences of maladaptive strategies, and develop more functional approaches to emotion regulation. Through its immersive and preventive design, emoWELL might support emerging adults in building emotional competencies that contribute to improved mental health and overall well-being.

### 2.5 Statistical analysis

SPSS version 28.0 and RStudio version 2024.04.1 + 748 were used to carry out the data analysis. To assess the intervention program’s impact, multivariate analyses of variance (MANOVA) were conducted to examine any initial differences between the experimental and control groups based on their pre-intervention scores. As previously mentioned in the participants section, differences were found between the control and experimental groups among the 135 resulting participants (Wilks’ lambda, *λ* = 0.80, *F* = 2.10, *p* < 0.001; *η*^2^ = 0.20). Therefore, Propensity Score Matching (PSM) was used to address these differences. PSM is a statistical technique designed to reduce bias by matching participants from the experimental group with those in the control group based on similar characteristics or probability of receiving the treatment. In this study, matching was performed using the following sociodemographic characteristics: gender, age, diagnosed physical or psychological difficulties, and whether participants were attending therapy. As a result of the matching process, an equal distribution of participants was obtained, with 50% in the control group and 50% in the experimental group. This natural balance achieved through PSM ensures greater comparability between the groups, enhancing the validity of the conclusions regarding the intervention’s effects (Gu and Rosenbaum, 1993; Li, 2013; Gnas et al., 2024).

Subsequently, multivariate analyses of covariance (MANCOVA) were applied to evaluate post-intervention changes in emotion regulation, with baselines scores included as covariates.

Hierarchical multiple regression analyses were conducted to assess the efficacy of emoWELL program. To quantify within-person changes in emotion regulation scales from pre- to post-intervention, the differences between T2 and T1 values were calculated. These difference scores were used as the dependent variables. The T1 score was included as a control variable in the initial step, followed by the experimental condition (coded as 1 for the experimental group and 0 for the control group) in the second step. After controlling pre-intervention scores, predictions based on the experimental condition indicate that significant changes can be attributed to the program.

To assess the impact of the intervention on different psychological dimensions, a Reliable Change Index (RCI) analysis was conducted. The RCI is used to determine whether changes in scores between pre- and post-intervention assessments are statistically reliable, helping to classify participants into categories based on their change status. Specifically, participants were categorized as having experienced an increase, decrease, or no change in their scores. Chi-square tests were then performed to examine the distribution of participants across these categories for both the control and experimental groups. This approach allowed us to compare the proportion of individuals showing significant improvement, deterioration, or stability within each group. These analyses provide insight into the effectiveness of the intervention by highlighting shifts in scores on various scales.

Since emoWELL was designed for a general emerging adulthood population, moderation analyses were conducted to examine the possible interaction between group (independent variable, experimental vs. control) and baseline depression and anxiety symptoms (introduced separately as moderating variables) on changes in the assessed variables (dependent variables) between the pre- and post-intervention.

Given that emoWELL does not directly address well-being, it was expected that the enhancement in emotion regulation resulting from the intervention would contribute to an improvement in well-being. Mediation analyses were conducted with the experimental condition as the independent variable, the change in total emotional dysregulation between pre- and post-intervention as the mediating variable, and the change in psychological well-being subscales between the two points (T1–T2) as the dependent variable.

Descriptive statistics were performed for the Likert-type question on how much they liked the intervention, ranging from 1 to 5. Qualitative and descriptive analyses were conducted for the open-ended question about what they liked most about the program.

## 3 Results

### 3.1 MANOVA and MANCOVA

The findings indicated that there were no statistically significant differences in the final sample between the experimental and control groups in the initial assessment (Wilks’ lambda, *λ* = 0.88, *F* = 0.86, *p* = 0.606; *η*^2^ = 0.12). Thus, both groups were comparable, as they had similar baseline levels (Table 1). The MANCOVA results showed statistically significant differences between experimental group and control group after the implementation of the program (Wilks’ λ = 68; *F* = 2.59; *p* = 0.003; *η*^2^ = 32).

**Table 1:** Impact of the program comparing emoWELL group and the control group.

Variables		emoWELL group	Control group	ANOVA		ANCOVA		
		*M*(SD)	*M*(SD)	*F*	*p*	*F*	*p*	η^2^
REAP	T1	30.94*(5.77)*	29.53*(4.63)*	2.10	0.150			
	T2	31.19*(4.53)*	28.97*(6.32)*			1.99	0.161	0.02
SUP	T1	13.67*(6.07)*	15.12*(5.07)*	1.94	0.167			
	T2	11.62*(5.47)*	14.65*(4.95)*			7.67^**^	0.007	0.07
ATT	T1	8.21*(2.99)*	9.44*(3.70)*	3.83	0.053			
	T2	7.98*(2.64)*	9.46*(3.56)*			0.85	0.358	0.01
CONF	T1	8.13*(2.67)*	8.28*(2.90)*	0.09	0.763			
	T2	8.11*(2.72)*	8.40*(3.08)*			0.27	0.605	0.00
REJ	T1	13.46*(5.86)*	14.54*(6.88)*	0.83	0.365			
	T2	12.51*(6.17)*	15.68*(4.08)*			8.35^**^	0.005	0.08
INTER	T1	11.47*(3.26)*	10.60*(4.54)*	1.40	0.239			
	T2	11.19*(3.94)*	10.98*(4.08)*			0.19	0.667	0.00
CONT	T1	16.56*(5.94)*	16.93*(7.51)*	0.08	0.772			
	T2	16.28*(6.27)*	18.84*(8.65)*			5.13^*^	0.026	0.05
TOTALDERS	T1	57.82*(15.18)*	59.79*(20.16)*	0.35	0.558			
	T2	56.07*(17.38)*	63.37*(21.88)*			5.61^*^	0.020	0.06
ACC	T1	16.18*(4.18)*	15.63*(4.29)*	0.47	0.494			
	T2	17.33*(3.92)*	16.56*(4.02)*			0.26	0.611	0.00
POS	T1	23.04*(5.48)*	23.19*(5.23)*	0.03	0.875			
	T2	23.54*(5.43)*	23.30*(4.96)*			0.18	0.669	0.00
AUT	T1	22.89*(4.53)*	22.96*(5.34)*	0.01	0.940			
	T2	23.51*(4.43)*	23.63*(4.94)*			0.08	0.774	0.00
ENV	T1	19.84*(4.55)*	20.33*(3.75)*	0.40	0.530			
	T2	20.39*(4.18)*	20.26*(3.37)*			0.00	0.973	0.00
PURP	T1	19.30*(4.35)*	19.09*(5.22)*	0.06	0.816			
	T2	20.39*(4.67)*	19.16*(4.83)*			2.78	0.099	0.03
PERS	T1	19.19*(3.26)*	18.77*(3.84)*	0.40	0.529			
	T2	19.95*(3.64)*	18.93*(3.84)*			1.18	0.280	0.01
DEPR	T1	6.12*(4.80)*	7.05*(5.13)*	1.00	0.320			
	T2	4.91*(4.55)*	6.05*(5.32)*			0.72	0.398	0.01
ANX	T1	5.68*(3.97)*	5.86*(4.75)*	0.05	0.831			
	T2	5.30*(4.48)*	5.40*(4.51)*			0.36	0.551	0.00

REAP = Cognitive reappraisal (*ERQ*); SUP = Emotional suppression (*ERQ*); ATT = Lack of emotional attention (*DERS*); CONF = Emotional confusion (*DERS*); REJ = Emotional rejection (*DERS*); INTER = Life interference (*DERS*); CONT = Emotional lack of control (*DERS*); TOTAL DERS = Emotion dysregulation global scale (*DERS*); ACC = Self-acceptance (*PWBS*); POS = Positive relations (*PWBS*); AUT = Autonomy (*PWBS*); ENV = Environmental mastery (*PWBS*); PURP = Purpose in life (*PWBS*); PERS = Personal Growth (*PWBS*); DEPR = Depression (*BSI*); ANX = Anxiety (*BSI*). M = Mean; SD = Standard deviation. η^2^ = Effect size (eta squared). ^*^p ≤ 0.05, ^**^p ≤ 0.01.

Specifically, differences between both groups were observed in emotional suppression (*F* = 7.67, *p* = 0.007), emotional rejection (*F* = 8.35, *p* = 0.005), emotional lack of control (*F* = 5.13, *p* = 0.026) and emotion dysregulation global scale (*F* = 5.61, *p* = 0.020), with reductions observed in the experimental group. The differences for purpose in life were marginally significant (*F* = 2.78, *p* = 0.099), showing a slight increase in the experimental group.

### 3.2 Multiple hierarchical regression

The multiple hierarchical regression assessed changes in emotion regulation strategies and emotion dysregulation (dependent variables) from pre- to post-intervention (T2-T1 difference scores), controlling for pre-intervention levels (T1 scores) and testing the effect of the experimental condition (experimental vs. control group).

In the first step of the analysis, the results indicated that the initial level significantly influenced the level of change (Table 2). Specifically, higher baseline scores were associated with less change in the outcome variables. As evidenced by negative beta coefficients, participants with higher initial scores showed less change, whereas those with lower baseline scores exhibited greater change. The second step showed that the experimental condition predicts change by controlling the baseline level of the variables. The higher the condition (1 > 0), the greater the change from T1 to T2 (positive regression coefficient). Specifically, the results indicated that emerging adults in the experimental group showed a reduction in the suppression of emotions, lack of emotional attention, emotional rejection, emotional lack of control, and global dysregulation (negative *β*).

**Table 2:** Hierarchical multiple regression analyses pre-intervention to follow-up.

Variables	Regression: Model 1				Regression: Model 2			
	*∆R^2^*	*∆F*	*β*	*t*	*∆R^2^*	*∆F*	*β*	*t*
REAP	0.14	18.67^***^	−0.38	−4.32^***^	0.02	2.49	0.14	1.58
SUP	0.15	20.26^***^	−0.39	−4.50^***^	0.07	9.68^**^	−0.26	−3.11^**^
ATT	0.19	25.61^***^	−0.43	−5.06^***^	0.02	2.42	−0.13	−1.56^***^
CONF	0.13	16.72^***^	−0.36	−4.08^***^	0.00	0.21	−0.04	−0.46
REJ	0.06	6.49^*^	−0.23	−2.55^*^	0.07	8.31^**^	−0.26	−2.88^**^
INTER	0.13	16.09^***^	−0.35	−4.01^***^	0.01	0.75	−0.08	−0.86
CONT	0.02	1.69	−0.12	−1.30	0.06	7.45^**^	−0.25	−2.73^**^
TOT	0.003	0.29	−0.05	−0.54	0.07	8.63^**^	−0.27	−2.94^**^
ACC	0.15	19.76^***^	−0.39	−4.45^***^	0.01	0.69	0.07	0.83
POS	0.12	15.02^***^	−0.34	−3.88^***^	0.00	0.45	0.06	0.67
AUT	0.16	21.54^***^	−0.40	−4.64^***^	0.00	0.02	−0.01	−0.13
ENV	0.18	24.95^***^	−0.43	−5.00^***^	0.01	0.84	0.08	0.92
PURP	0.14	18.50^***^	−0.38	−4.30^***^	0.02	3.08	0.15	1.75
PERS	0.10	12.46^***^	−0.32	−3.53^***^	0.02	2.01	0.13	1.42
DEPR	0.13	16.19^***^	−0.36	−4.02^***^	0.00	0.52	−0.06	−0.72
ANX	0.15	20.27^***^	−0.39	−4.50^***^	0.00	0.000	0.002	0.02

REAP = Cognitive reappraisal (*ERQ*); SUP = Emotional suppression (*ERQ*); ATT = Lack of emotional attention (*DERS*); CONF = Emotional confusion (*DERS*); REJ = Emotional rejection (*DERS*); INTER = Life interference (*DERS*); CONT = Emotional lack of control (*DERS*); TOT = Total Scale (DERS); ACC = Self-acceptance (*PWBS*); POS = Positive relations (*PWBS*); AUT = Autonomy (*PWBS*); ENV = Environmental mastery (*PWBS*); PURP = Purpose in life (*PWBS*); PERS = Personal Growth (*PWBS*); DEPR = Depression (*BSI*); ANX = Anxiety (*BSI*); ∆*R*^2^ = Increment of *R*^2^; ∆*F* = Increment of *F*; β = Standardized coefficient; *t* = *t*-value.

^*^p ≤ 0.05, ^**^p ≤ 0.01, ^***^p ≤ 0.001.

Model 1: predictor = pre-intervention score; Model 2: predictor = experimental condition, controlled for pre-intervention score; outcome variables: change scores pre-intervention to follow-up were used for each for the regression analyses. Standardized beta values were reported.

### 3.3 Reliable change index (RCI)

The calculation of the RCI was used to classify participants into categories based on their reliable change status. Specifically, participants were categorized as having experienced an increase, decrease, or no change in their scores (Table 3).

**Table 3:** Reliable change index pre-intervention to post-intervention.

Variables	*χ* ^2^	emoWELL group			Control group		
		RC-I *n*(*%*)	WRC *n*(*%*)	RC-D *n*(*%*)	RC-I *n*(*%*)	WRC *n*(*%*)	RC-D *n*(*%*)
REAP	2.64	3*(5.3)*	51*(89.5)*	3*(5.3)*	1*(1.8)*	49*(86)*	7*(12.3)*
SUP	3.63	1*(1.8)*	48*(84.2)*	8*(14)*	0*(0)*	54*(94.7)*	3*(5.3)*
ATT	0.77	2*(3.5)*	49*(86)*	6*(10.5)*	4*(7)*	48*(84.2)*	5*(8.8)*
CONF	1.88	3*(5.3)*	50*(87.7)*	4*(7)*	*3(3.5)*	53*(93)*	1*(1.8)*
REJ	5.81^*^	2*(3.5)*	52*(91.2)*	3*(5.3)*	*9(15.8)*	43*(75.4)*	5*(8.8)*
INTER	2.94	6*(10.5)*	41*(71.9)*	10*(17.5)*	*7(12.3)*	46*(80.7)*	4*(7)*
CONT	2.29	4*(7)*	50*(87.7)*	3*(5.3)*	9*(15.8)*	46*(80.7)*	2*(3.5)*
TOT	1.89	6*(10.5)*	46*(80.7)*	5*(8.8)*	9*(15.8)*	46*(80.7)*	2*(3.5)*
ACC	1.51	5*(8.8)*	52*(91.2)*	0*(0)*	3*(5.3)*	53*(93)*	1*(1.8)*
POS	0.44	3*(5.3)*	51*(89.5)*	3*(5.3)*	2*(3.5)*	53*(93)*	*2(3.5)*
AUT	1.01	0*(0)*	57*(100)*	0*(0)*	*1(1.8)*	56*(98.2)*	0*(0)*
ENV	1.33	2*(3.5)*	55*(96.5)*	0*(0)*	1*(1.8)*	55*(96.5)*	1*(1.8)*
PURP	0.44	5*(8.8)*	51*(89.5)*	1*(1.8)*	4*(7)*	51*(89.5)*	2*(3.5)*
PERS	3.43	4*(7)*	52*(91.2)*	1*(1.8)*	6*(10.5)*	46*(80.7)*	5*(8.8)*
DEPR	0.33	3*(5.3)*	48(84.2)	6*(10.5)*	2*(3.5)*	50*(87.7)*	5*(8.8)*
ANX	0.12	4*(7)*	48(84.2)	5*(8.8)*	5*(8.8)*	47*(82.5)*	5*(8.8)*

RC = reliable change-increase; WRC = without reliable change; NRC = reliable change-decrease. *n*% = number of participants and corresponding percentage within each category. REAP = Cognitive reappraisal (*ERQ*); SUP = Emotional suppression (*ERQ*); ATT = Lack of emotional attention (*DERS*); CONF = Emotional confusion (*DERS*); REJ = Emotional rejection (*DERS*); INTER = Life interference (*DERS*); CONT = Emotional lack of control (*DERS*); TOT = Total Scale (DERS); ACC = Self-acceptance (*PWBS*); POS = Positive relations (*PWBS*); AUT = Autonomy (*PWBS*); ENV = Environmental mastery (*PWBS*); PURP = Purpose in life (*PWBS*); PERS = Personal Growth (*PWBS*); DEPR = Depression (*BSI*); ANX = Anxiety (*BSI*). ^*^p ≤ 0.05.

Based on the four subscales identified as significant by the ANCOVA analysis – emotional suppression, emotional rejection, emotional lack of control, and the emotion dysregulation global scale – the cross-tabulation analysis identified emotional rejection as the only variable showing a significant change. Specifically, 15.8% of participants in the control group experienced an increase in terms of reliable change compared to the experimental group.

For the remaining scales, although not significantly, 14% of participants in the experimental group showed a decrease in emotional suppression relative to the control group. The control group also showed a greater increase in emotional lack of control (15.8%) and in the emotion dysregulation global scale (15.8%) compared to the experimental group’s levels for emotional lack of control (7%) and the emotion dysregulation global scale (10.5%).

Despite the previous regression models indicated a reduction in levels of lack of emotional attention, the RCI distribution was very similar in both groups. Regarding other scales, although not statistically significant, 17.5% of participants in the experimental group showed a reduction in emotion life interference levels, compared to 7% in the control group.

### 3.4 Moderating effects of depression and anxiety

Moderation analyses tested whether baseline depression and anxiety symptoms (moderators) influenced the relationship between group (independent variable: experimental vs. control) and changes in the assessed variables (dependent variables) from pre- to post-intervention (Table 4).

**Table 4:** Moderating effects on the intervention program.

T1–T2 change		Depression	Anxiety
Cognitive reappraisal	*b*	−0.01	−0.24
	*t*	−0.03	−1.17
	95% CI	−0.37, −0.35	−0.65, 0.17
Emotional suppression	*b*	0.00	−0.03
	*t*	0.01	−0.20
	95% CI	−0.28, 0.29	−0.36, 0.30
Lack of emotional attention	*b*	0.18	0.13
	*t*	2.03^*^	1.20
	95% CI	0.00, 0.37	−0.08, 0.34
Emotional confusion	*b*	0.12	0.21
	*t*	1.34	2.14*
	95% CI	−0.06, 0.29	0.02, 0.41
Emotional rejection	*b*	0.08	0.07
	*t*	0.48	0.38
	95% CI	−0.25, 0.41	−0.31, 0.45
Life interference	*b*	0.24	−0.03
	*t*	2.22^*^	−0.22
	95% CI	0.03, 0.45	−0.28, 0.23
Emotional lack of control	*b*	0.24	0.17
	*t*	1.47	0.88
	95% CI	−0.08, 0.57	−0.21, 0.55
Total scale	*b*	0.86	0.55
	*t*	2.37^*^	1.29
	95% CI	0.14, 1.59	−0.30, 1.40
Self-acceptance	*b*	−0.12	−0.06
	*t*	−1.35	−0.52
	95% CI	−0.31, 0.06	−0.27, 0.16
Positive relations	*b*	−0.14	−0.12
	*t*	−1.12	−0.85
	95% CI	−0.37, 0.10	−0.39, 0.16
Autonomy	*b*	−0.27	−0.02
	*t*	−2.30^*^	−0.14
	95% CI	−0.51, −0.04	−0.30, 0.26
Environmental mastery	*b*	−0.03	−0.07
	*t*	−0.22	−0.56
	95% CI	−0.25, 0.20	−0.33, 0.18
Purpose in life	*b*	−0.35	−0.27
	*t*	−2.67^**^	−1.77
	95% CI	−0.61, −0.09	−0.58, 0.03
Personal Growth	*b*	−0.10	−0.11
	*t*	−0.96	−0.86
	95% CI	−0.31, 0.11	−0.35, 0.14

CI = confidence interval; b refers to the unique contribution of the interaction term (moderator × group) to the prediction of the change score after controlling for the separate effects of group and moderator. ^*^p ≤ 0.05, ^**^p ≤ 0.01.

**Figure 2:**
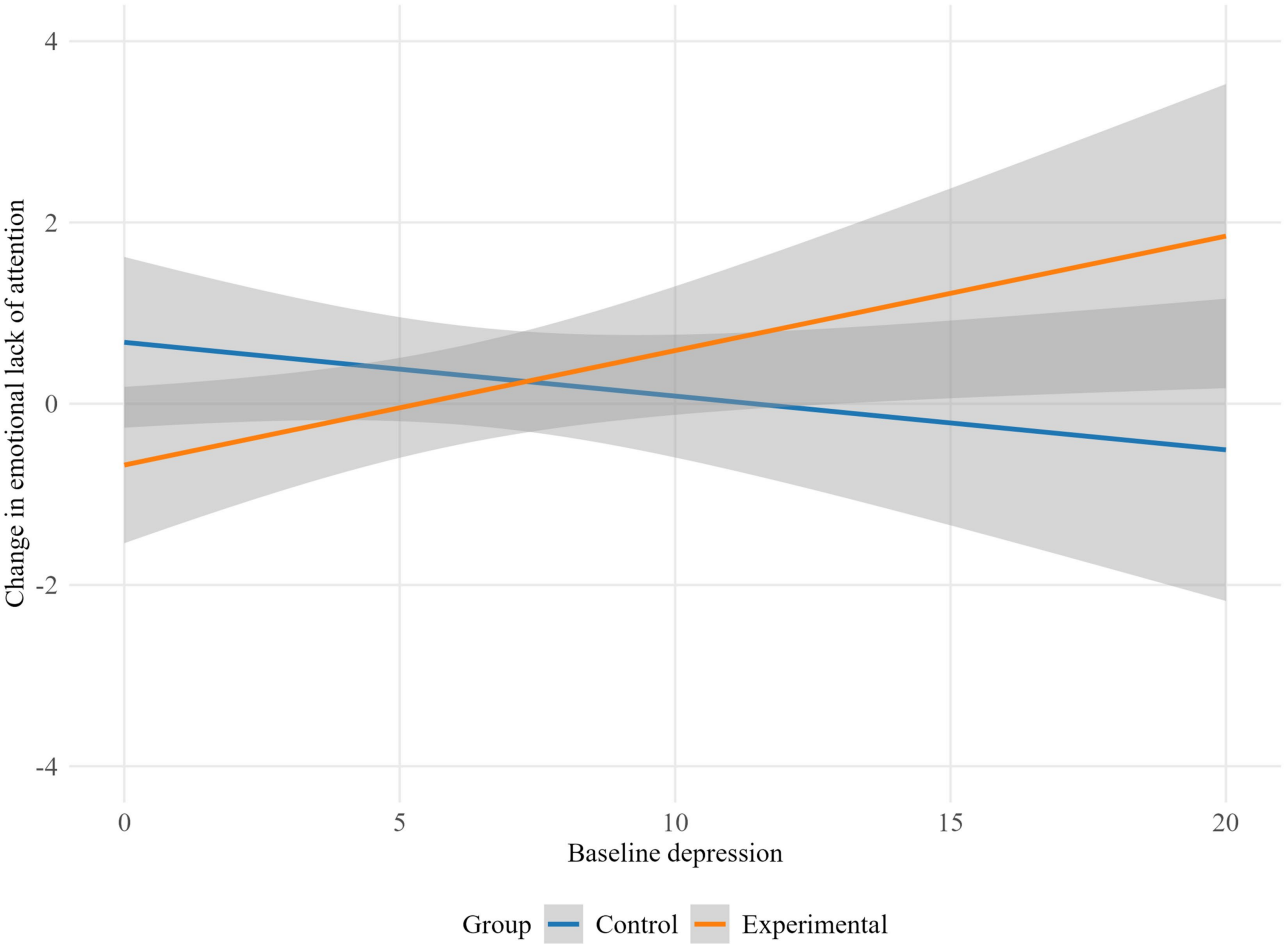
Moderation effect of baseline depression on lack of emotional attention by group.

**Figure 3:**
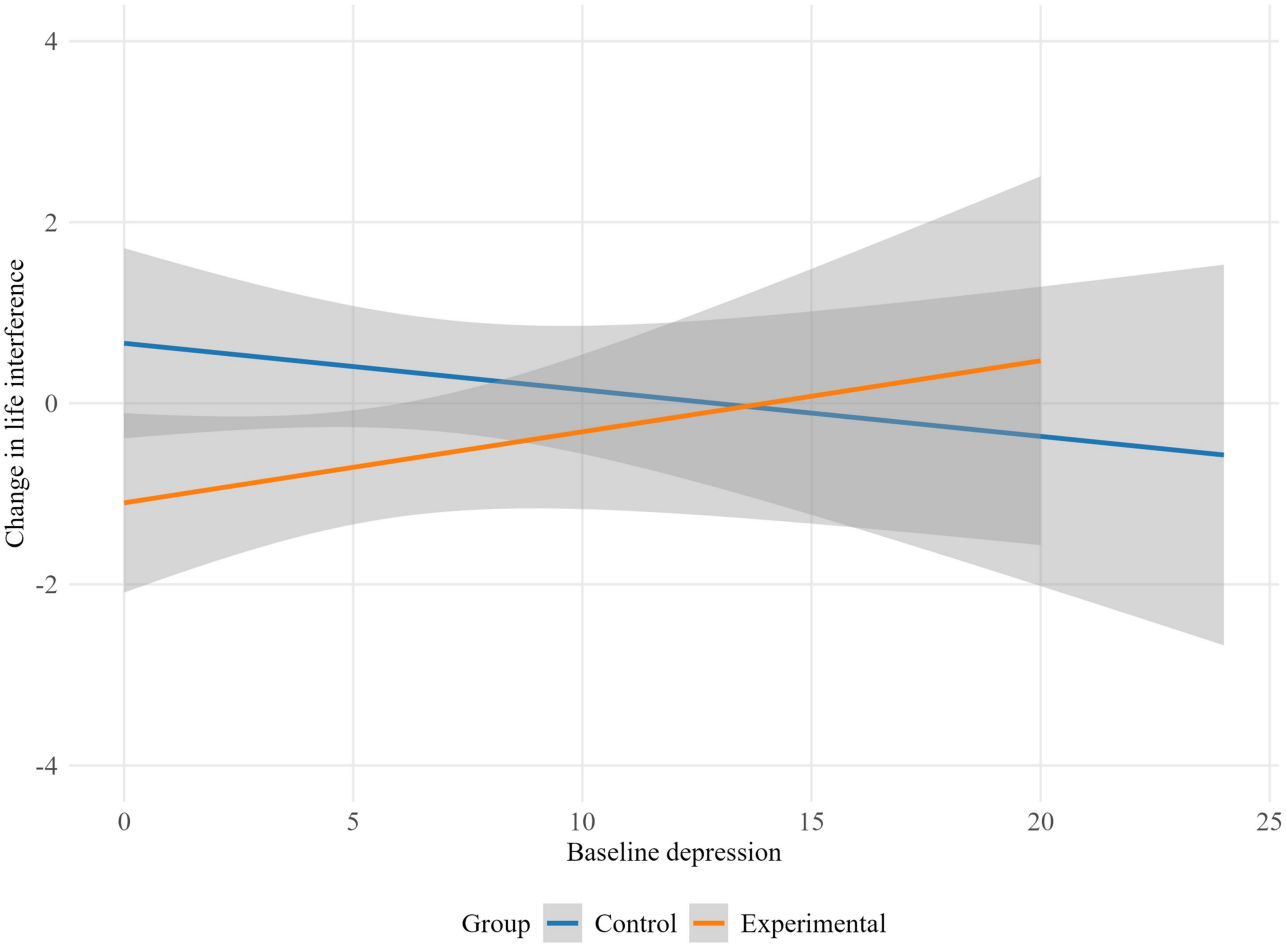
Moderation effect of baseline depression on life interference by group.

**Figure 4:**
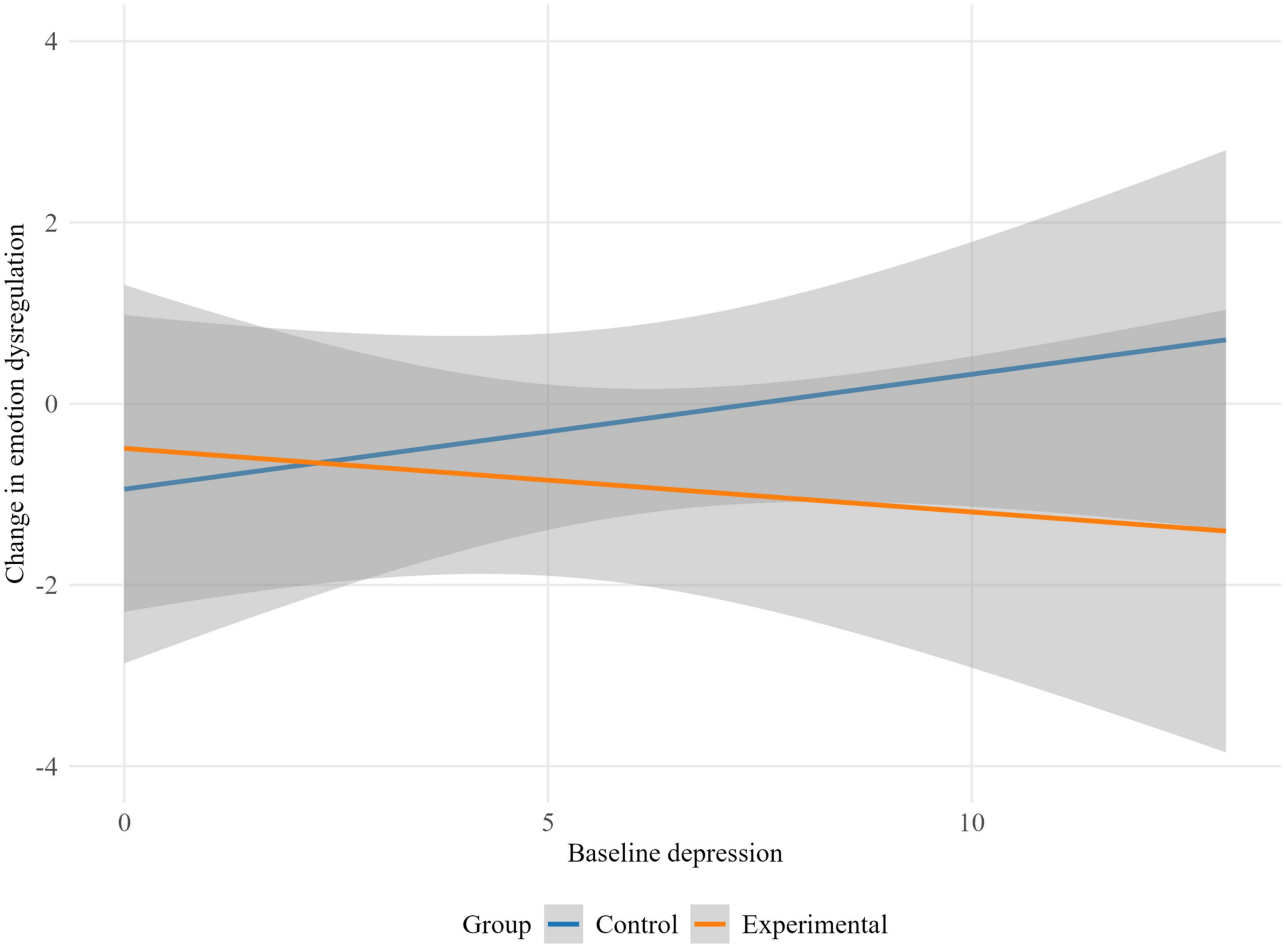
Moderation effect of baseline depression on emotion dysregulation global scale by group.

The results indicate that depression moderated the influence of the intervention on lack of emotional attention (*p* = 0.045), life interference (*p* = 0.029), emotion dysregulation global scale (*p* = 0.019), autonomy (*p* = 0.023) and purpose in life (*p* = 0.009). Specifically, the experimental group showed higher emotional lack of attention and life interference scores as baseline depression increased, while the control group displayed a decrease in emotional lack of attention and life interference with higher baseline depression levels (Figures 2, 3). Conversely, in the control group, higher baseline depression levels were associated with an increase in overall emotion dysregulation. Meanwhile, in the experimental group, higher baseline depression was linked to a decrease in overall emotion dysregulation (Figure 4).

**Figure 5:**
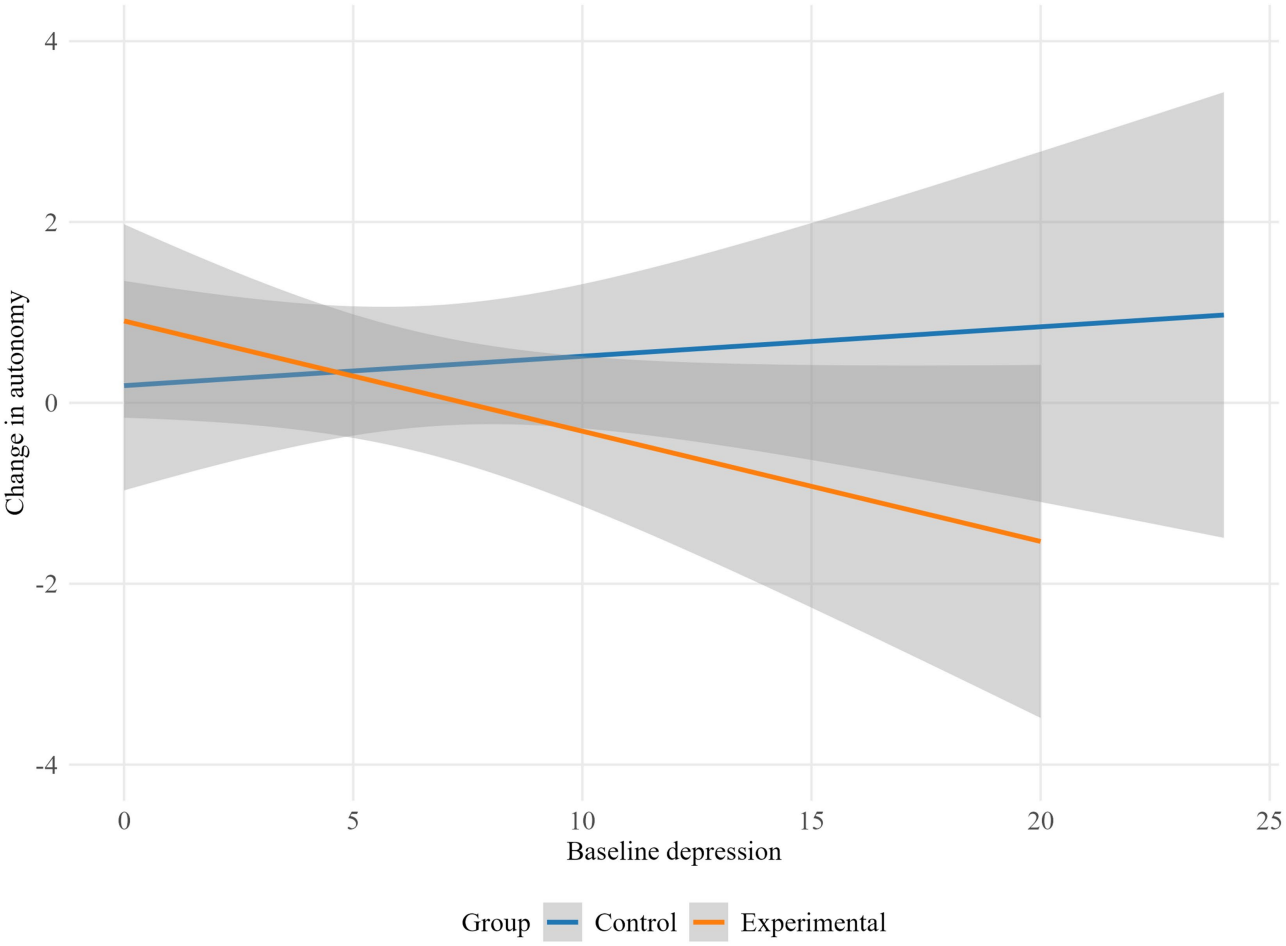
Moderation effect of baseline depression on autonomy by group.

**Figure 6:**
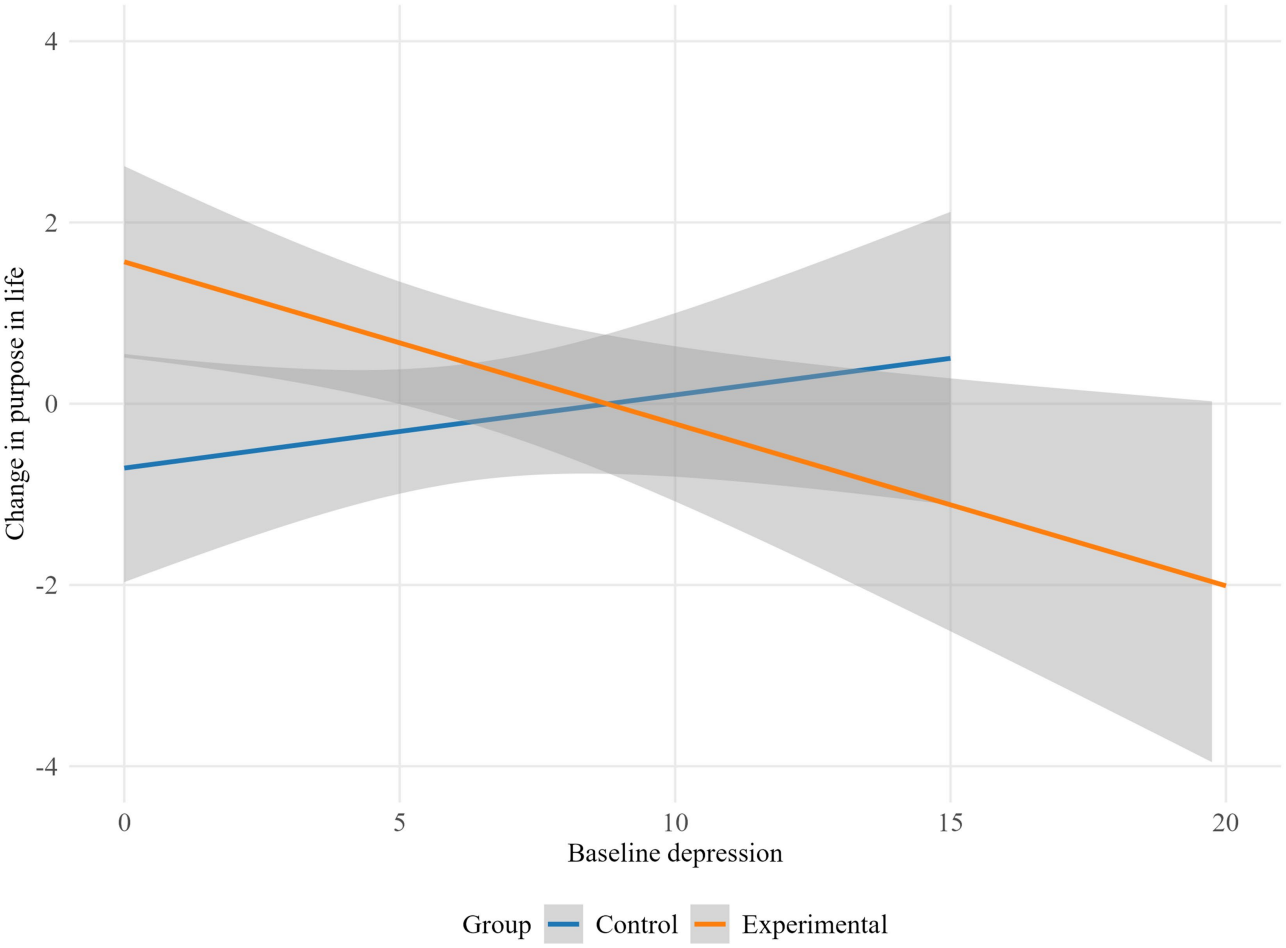
Moderation effect of baseline depression on purpose in life by group.

Regarding psychological well-being, for participants with low baseline depression levels, the experimental group showed an increase in autonomy and purpose in life, while the control group exhibited minimal change. However, for those with high baseline depression, the pattern reversed: the experimental group demonstrated a decrease in autonomy and purpose in life, whereas the control group showed a slight improvement (Figures 5, 6).

**Figure 7:**
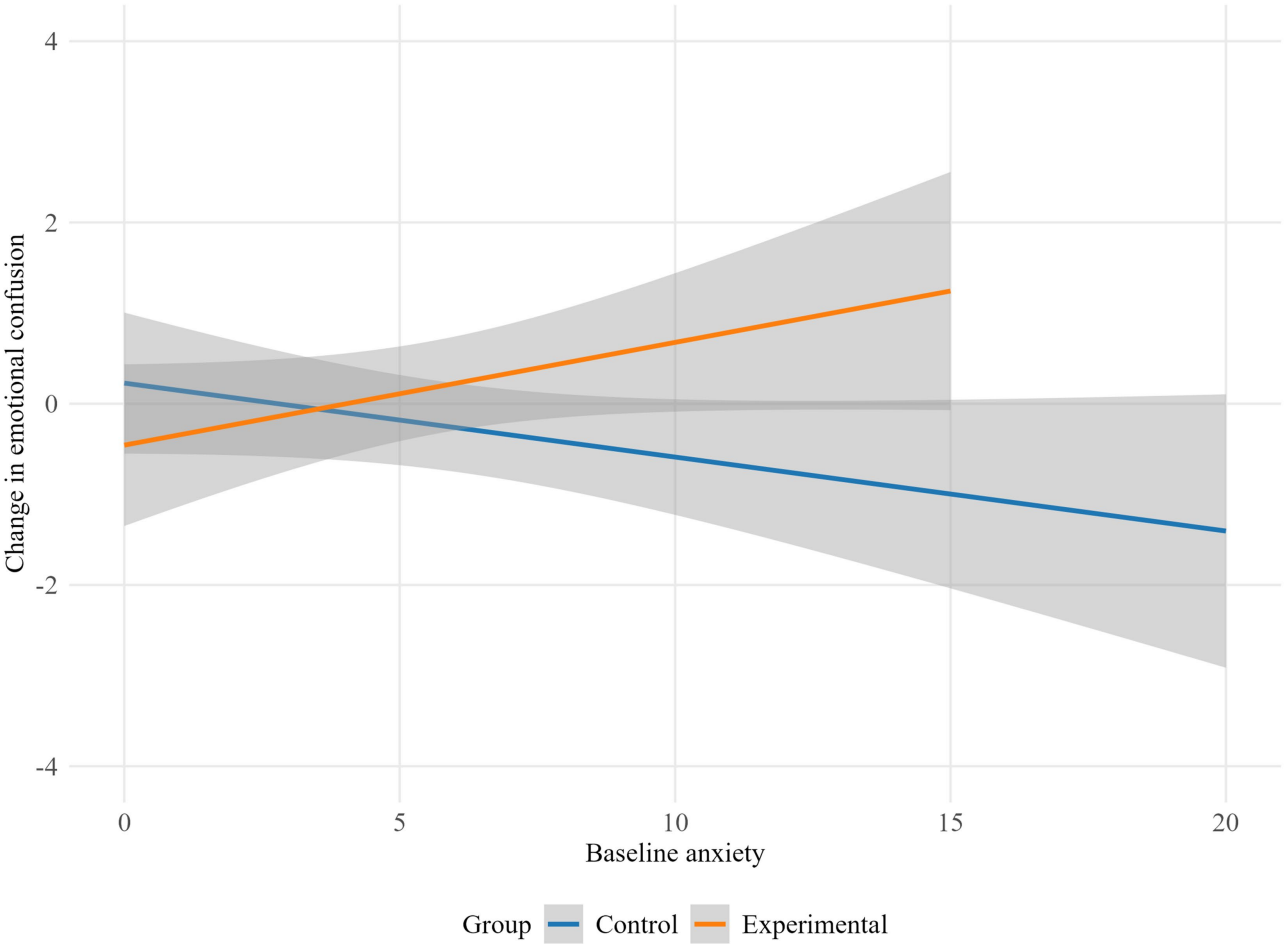
Moderation effect of baseline anxiety on emotional confusion by group.

Anxiety levels moderated the intervention influence on emotional confusion (*p* = 0.034). As shown in Figure 7, the experimental group displayed increased emotional confusion as baseline anxiety levels rose, while the control group showed decreased emotional confusion with higher baseline anxiety.

### 3.5 Mediating effects of emotion dysregulation on well-being

Mediation analyses were employed to assess whether the change in total emotional dysregulation (mediating variable) influenced the relationship between the group condition (independent variable, experimental vs. control) and the change in psychological well-being subscales (dependent variables) from pre- to post-intervention (T1–T2) (Table 5). Statistically significant indirect effects were found for self-acceptance (*p* = 0.014) and environmental mastery (*p* = 0.015), and a marginally significant effect was observed for purpose in life (*p* = 0.062). However, no significant direct or total effects were found for any of these three well-being subscales.

**Table 5:** Mediation analysis: effect of change in emotion dysregulation on changes in psychological well-being.

T1–T2 change in well-being	B ACME	95% CI ACME	B ADE	95% CI ADE	B Total effect	95% CI Total effect	B Proportion mediated	95% CI Proportion mediated
Self-acceptance	0.23^*^	0.04, 0.52	−0.01	−0.85, 0.89	0.23	−0.62, 1.15	1.03	−6.75, 6.85
Positive relations	0.17	−0.22, 0.59	0.23	−0.95, 1.43	0.40	−0.79, 1.55	0.42	−3.64, 4.94
Autonomy	0.21	−0.13, 0.70	−0.26	−1.48, 0.94	−0.05	−1.24, 1.11	−3.95	−6.22, 7.12
Environmental mastery	0.46^*^	0.05, 1.06	0.15	−0.88, 1.15	0.61	−0.44, 1.69	0.75	−5.38, 6.47
Purpose in life	0.47	−0.01, 1.15	0.55	−0.92, 2.06	1.02	−0.26, 2.34	0.46	−3.07, 4.05
Personal growth	0.07	−0.23, 0.42	0.52	−0.55, 1.61	0.60	−0.44, 1.62	0.12	−1.87, 2.13

B = unstandardized regression coefficient; ACME = average causal mediation effect; ADE = average direct effect. *p ≤ 0.05. Bootstrapped confidence intervals based on 5,000 samples.

### 3.6 Program assessment by participants

Participants from the experimental group were asked to assess how much they liked the emoWELL program, with responses ranging from 1 (I did not like it at all) to 5 (I liked it a lot). The distribution of responses was as follows: 0% of participants selected “1,” 5.3% selected “2,” 17.5% selected “3,” 63.2% selected “4,” and 14% selected “5.”

In relation to the open question (What did you like most about emoWELL?), the responses were grouped into three general categories (Table 6).

**Table 6:** Features participants liked most about emoWELL.

Categories	Subcategories	NR in emoWell group
General game assessment	Dynamic and original	7 (6.67%)
	Simple to understand and play	4 (3.81%)
	Didactic and useful	13 (12.38%)
	Entertaining and funny	9 (8.57%)
	Interesting and intriguing	6 (5.71%)
	Subtotal	39 (37.14%)
Game elements assessment	Story	5 (4.76%)
	Esthetics and scenarios	5 (4.76%)
	Characters	3 (2.86%)
	Specific activities	4 (3.81%)
	Subtotal	17 (16.19%)
Psychological content assessment	Psychoeducation on emotions and emotion regulation	22 (20.95%)
	Introspection, awareness, and identification of emotions	10 (9.52%)
	Emotional understanding	8 (7.62%)
	Implementation of emotion regulation strategies	7 (6.67%)
	Other benefits	2 (1.90%)
	Subtotal	49 (46.66%)
TOTAL		105 (100%)

NR, number of references.

The first category, general game assessment, refers to overall opinions about the game, where key features of serious games are also highlighted. Specifically, the game was described as dynamic and original (e.g., “I liked that it feels like a ‘normal’ game, similar to the ones we played as kids, which makes it very dynamic.,” “The innovative way it uses a tool to improve socio-emotional skills.”), simple to understand and play (e.g., “It was easy to do.,” “The game helps you understand everything very well.”), didactic and useful (e.g., “It’s educational.,” “It helps you learn concepts.,” “It’s a very useful tool.”), entertaining and funny (e.g., “I found it entertaining.”), and interesting and intriguing (e.g., “The entire story seemed quite interesting to me.”). In this category, the subcategory didactic and useful stood out, with a total of 12.38% of the responses.

The second category, game elements assessment, encompasses opinions about different aspects of the game, such as the story (e.g., “The story was well-constructed and cohesive, it was appealing.”), esthetics and scenarios (e.g., “What I liked most about emoWELL were the different scenarios.,” “The game’s aesthetics.”), characters (e.g., “The characters.”), and specific activities (e.g., “What I liked the most were the meditations.,” “I liked the fact that you could write like in a journal”). In this category, the story and esthetics and scenarios received the most responses (4.76% each).

Finally, the third category, psychological content assessment, includes various opinions regarding the learning related to emotion regulation. Some participants highlighted theoretical learning about emotions and emotion regulation, or provided general feedback about learning, which was captured in the subcategory psychoeducation on emotions and emotion regulation (e.g., “What I liked the most is that you learn a lot without realizing it.,” “I’ve learned about the importance of emotions and how to manage them,” “I have learned about emotion regulation strategies.”). Others reported improvements in introspection, awareness, and identification of emotions (e.g., “It’s pretty good because it makes you reflect on yourself.,” “I’ve learned to identify some emotions.”) and better emotional understanding (e.g., “It helps you understand emotions.”). Some participants mentioned that they had begun using more adaptive regulation strategies and/or stopped using dysfunctional regulation strategies, captured in the subcategory implementation of emotion regulation strategies (e.g., “I think what I liked the most – and in fact I’ve started applying it in my daily life – is how useful the emphasis on emotional self-management mechanisms was. Since they were defined so clearly and accessibly, and provided examples, I was able to understand and identify the ones I use, as well as try to develop the ones that are beneficial to me.”). Lastly, others noted other benefits (e.g., “It helps you strengthen your patience.”). For this category, the responses highlighted the subcategory psychoeducation on emotions and emotion regulation (20.95% of the responses).

## 4 Discussion

Previous research has highlighted the importance of having optimal emotion regulation for better mental health adjustment in emerging adults (Newman and Nezlek, 2022; Zagaria et al., 2023; Gatto et al., 2022). Considering the use of video games during this life stage and the effectiveness of serious games on emotion regulation (Micallef et al., 2021; Rodrigo-Yanguas et al., 2023), the present study aimed to evaluate the effectiveness of a serious game designed to improve emotion regulation in emerging adults.

Evaluating the original hypotheses against the findings reveals varying levels of support across the predictions. The results demonstrate a more nuanced picture than initially proposed, particularly regarding which specific components of emotion regulation were affected by the intervention, which dimensions of psychological well-being showed improvement through mediation, and how baseline emotional symptoms moderated program effectiveness in sometimes unexpected ways.

Regarding the first hypothesis, an improvement in emotion regulation was expected. Specifically, a reduction in emotion dysregulation was anticipated, meaning fewer difficulties in emotion regulation, a decrease in expressive suppression, and an increase in cognitive reappraisal.

ANCOVA results revealed relevant changes after the intervention in the group that played emoWELL in four key aspects: expressive suppression, emotional rejection, emotional lack of control and general emotion dysregulation. Multiple hierarchical regression analyses allowed direct quantification of how much change in scores was attributed to the intervention program. This analysis reinforced the previous findings, showing decreases in expressive suppression, rejection to emotions, problems with the control of emotions and global emotion regulation difficulties, and also indicating fewer difficulties in paying attention to emotions in the experimental group following the intervention. When identifying what percentage of people responded positively or negatively in each group, the Reliable Change Index (RCI) showed statistical significance only for rejection to emotions. Emerging adults in the control group had a greater increase in their emotion rejection compared to those who played emoWELL. While not statistically significant, the distribution of these changes also suggests that participants in the experimental group indicated a trend toward a greater reduction in their expressive suppression, emotion confusion, and emotion life interference. In this line, emoWELL participants also showed a tendency to increase their cognitive reappraisal strategy, while control group participants reported a decrease in this strategy in the post-intervention assessment. Furthermore, emerging adults in the experimental group also tended to show smaller increases in their emotional control problems and difficulties with overall emotion regulation.

Based on these results, our hypotheses are partially supported. By using the emoWELL program, emerging adults seem to tend to use less expressive suppression strategy and experience less emotional dysregulation. Specifically, it seems particularly effective in preventing an increase in rejection of emotions, as well as halting the growth of emotional control difficulties and overall difficulties in emotion regulation. These findings align with previous research that highlight the importance of reducing maladaptive strategies such as expressive suppression during emerging adulthood (John and Gross, 2004). The reduction in expressive suppression and the improvement in emotional dysregulation appear especially significant, as these variables have been linked to negative outcomes for overall health and well-being (Cheung et al., 2021; Newman and Nezlek, 2022; Rufino et al., 2022). Nonetheless, no significant differences were observed in the various analyses for cognitive reappraisal, nor for the confusion or interference of emotions in daily life. This could be explained by the psychoeducational component of the program, as evidenced by the qualitative evaluation of the participants, who emphasized the psychoeducational content of the platform and the help it provided in raising awareness and learning to identify their emotions. Serious games that address emotion regulation often include many psychoeducational elements (Silva et al., 2024; Heng et al., 2023). Acquiring more information may have helped the emerging adult participants gain more introspection, ceasing to reject emotions and their expression inappropriately, starting to no longer experience them as overwhelming. However, although various practices and activities are worked on in the game to implement adaptive emotion regulation strategies, future adjustments may be necessary to continue training these skills to a greater extent, subsequently observing the potential impact on other elements of emotion regulation, such as cognitive reappraisal.

Regarding the second hypothesis, it was expected that psychological well-being would improve indirectly through the enhancement of emotion regulation. Although no direct effects on psychological well-being were observed, the mediation analyses revealed significant indirect effects through the improvement in emotion regulation, specifically in the dimensions of self-acceptance and environmental mastery. These results are consistent with previous research linking adaptive emotion regulation with various indicators of well-being (Mouatsou and Koutra, 2021). When individuals reduce emotional suppression and rejection, they might develop a more accepting attitude toward their experiences, while enhanced emotional control could enable better management of environmental demands. The absence of indirect effects on other dimensions (positive relations, autonomy, purpose in life, and personal growth) might be due to these aspects encompassing skills beyond emotion regulation, such as specific social abilities, independent decision-making, goal-setting, and continuous personal development, which could require more targeted interventions.

In this regard, it was also intended to explore whether anxious and depressive symptoms constituted a limitation in working on emotion regulation. ANCOVA analyses and regressions showed no improvement in anxiety and depression with the program. A moderation analysis indicated that groups with higher previous depression levels showed fewer difficulties overall in their emotion dysregulation by the end of the program. However, other moderation analyses indicated that high levels of depression prior to playing emoWELL led to greater difficulties in attending to emotions and a greater perception that emotions cause interference in daily performance. Greater depression levels also seem to cause a reduction in autonomy after playing emoWELL, with participants also perceiving more difficulties in achieving future goals. Finally, the baseline anxiety favored that emerging adults who played emoWELL after the program had more difficulty perceiving how they felt. This is consistent with part of the literature, which indicates that psychological disorders can interfere with adaptive emotion regulation (Aldao, 2013; Visted et al., 2018). EmoWELL promotes introspection through psychoeducation, and introspection plays a complex and ambivalent role in the subjective well-being of emerging adults. While for some emerging adults it is important to develop introspection for good mental health, for others it may be harmful, depending on factors such as the environment or personal goals (Zhu et al., 2024; Herwig et al., 2018). Introspection involves focusing on one’s own cognitions and feelings, so a high level of introspection could negatively affect current health and adaptability, where some people who pay less attention to their emotions tend to perceive themselves as less stressed (Maio-Esteves, 1990; De La Barrera et al., 2023). Therefore, when addressing emotional states related to previous psychopathological symptoms through technological platforms, it is important to ensure appropriate psychological support is provided. emoWELL has been specifically designed to target emotion regulation skills in the general population of emerging adults, with indirect benefits expected for psychological well-being, rather than as a direct intervention for anxiety or depression. While the program shows promise for individuals with low levels of emotional symptoms, participants with greater anxiety and depression symptoms may need additional professional support to fully benefit from the emotion regulation training. emoWELL is thus most appropriately used as a complementary tool under professional supervision or in university settings with appropriate support, focusing on enhancing emotion regulation abilities rather than as a direct intervention for existing mood or anxiety disorders.

These findings underscore the importance of implementing a formal screening process before recommending serious games like emoWELL to emerging adults. The program may be beneficial for those without significant emotional symptoms who are seeking to preventively enhance their emotion regulation competencies. However, individuals with elevated anxiety or depression symptoms should first receive conventional psychological support. University mental health services and other professionals should conduct proper assessments to identify those who might experience adverse effects from self-directed emotion regulation programs. Once these individuals have achieved symptom reduction through appropriate psychological intervention, they could then benefit from emoWELL as a complementary tool. This staged approach acknowledges an important ethical distinction: although emotion regulation can protect against depressive symptoms in this stage (Roos and Bennett, 2023), there is a substantial difference between prevention and intervention for existing clinical symptoms. Digital interventions should be positioned within a broader mental health framework that recognizes when technology-based tools are appropriate and when more intensive professional support is required. Implementing such screening protocols would not only enhance the effectiveness of emoWELL but would also mitigate potential risks, ensuring that emerging adults receive the appropriate level of support based on their individual psychological needs.

In summary, emoWELL is a preventive tool for emerging adulthood that works on emotion regulation, indirectly promoting well-being. The qualitative evaluation of the program revealed high satisfaction among participants, highlighting characteristics of serious games such as their didactic and entertaining nature, as well as the learning elements of the platform (Din et al., 2023). Although the present study has advantages, it also has limitations. The relatively small sample size and the absence of long-term follow-up limit the generalization of the results. Moreover, the sample had a significant overrepresentation of women (86.8%) compared to men (13.2%). Although participants were evenly distributed between the experimental and control groups, the limited gender diversity represents an important limitation for the generalizability of the findings. The lack of interrater agreement in the qualitative analysis suggests that this part of the results should be interpreted with caution. Future research should include more diverse samples of emerging adults, longer follow-up periods, and more robust evaluations of the qualitative components. Future research should also aim to include more balanced gender representation and actively include participants across the gender spectrum, not limited to binary gender identities, to determine whether the effects of emoWELL on emotion regulation are consistent across different gender experiences and identities. Although the hierarchical multiple regression analysis revealed some significant changes in emotion regulation outcomes attributable to the emoWELL program, the effect sizes observed were relatively small. This suggests that the intervention’s impact may be modest, and the overall efficacy could be enhanced with adjustments in future studies. Additionally, the sample, which is mainly composed of university students from a single geographic area, does not allow for extrapolation of the findings to other profiles of emerging adults. The lack of interrater agreement in the qualitative analysis suggests that this part of the results should be interpreted with caution. Future research should include more diverse samples of emerging adults, longer follow-up periods, and more robust evaluations of the qualitative components. Furthermore, it is important to consider the pre-existing levels of anxiety and depression in emerging adults in order to identify those with low levels before playing emoWELL and exclude those with greater levels. It would also be interesting in future studies with samples matched for low anxiety and depression symptoms to examine potential changes in these variables after the intervention.

Beyond the theoretical contributions, this study has several practical implications for real-world applications. First, emoWELL could be integrated into university counseling services as a preventive tool, providing students with a self-directed resource for developing emotion regulation skills before critical periods like exams or transitions. Educational institutions could incorporate this serious game as a supplementary resource in psychology and mental health. Second, the findings regarding the moderating effect of baseline depression and anxiety levels have direct implications for implementation: institutions should consider screening for emotional symptoms before recommending the tool, ensuring appropriate professional support for those with elevated symptoms. Third, the game’s digital format allows for scalable, cost-effective dissemination across various educational settings, addressing the growing demand for accessible mental health resources for emerging adults. The positive user feedback regarding the psychoeducational content suggests that emoWELL could serve as a practical introduction to emotion regulation concepts, potentially increasing emotional literacy and reducing stigma around seeking further support. Finally, the game’s framework could serve as a model for developing similar tools targeting other psychological competencies relevant to this developmental stage, such as social skills, creating a comprehensive digital toolkit for emerging adult wellbeing in educational contexts.

To conclude, this preliminary study demonstrates that emoWELL is a promising tool for improving emotion regulation in emerging adults. Its serious game format and psychoeducational approach seem to facilitate the learning and practice of emotion regulation skills.

## Data Availability

The raw data supporting the conclusions of this article will be made available by the authors, without undue reservation.
